# Striking a Balance: A Qualitative Study of Next of Kin Participation in the Care of Older Persons in Nursing Homes in Sweden

**DOI:** 10.3390/healthcare6020046

**Published:** 2018-05-11

**Authors:** Birgitta Wallerstedt, Lina Behm, Åsa Alftberg, Anna Sandgren, Eva Benzein, Per Nilsen, Gerd Ahlström

**Affiliations:** 1 Center for Collaborative Palliative Care, Department of Health and Caring Sciences, Faculty of Health and Life Sciences, Linnaeus University, SE-351 95 Växjö, Sweden; birgitta.wallerstedt@lnu.se (B.W.); anna.sandgren@lnu.se (A.S.); eva.benzein@lnu.se (E.B.); 2 Department of Health Sciences, Faculty of Medicine, Lund University, SE-221 00 Lund, Sweden; lina.behm@med.lu.se; 3 Department of Social Work, Faculty of Health and Society, Malmö University, SE-205 06 Malmö, Sweden; asa.alftberg@mah.se; 4 Department of Medical and Health Sciences, Linköping University, SE-581 83 Linköping, Sweden; per.nilsen@liu.se

**Keywords:** end-of-life care, family member, involvement, life-limiting disease, next of kin, palliative care, participation, sheltered housing, significant others, relatives

## Abstract

Most of the care in nursing homes is palliative in nature, as it is the oldest and the frailest people who live in nursing homes. The aim of this study was to explore next of kin’s experiences of participating in the care of older persons at nursing homes. A qualitative design was used, based on semi-structured interviews with 40 next of kin, and analyzed using qualitative content analysis. An overarching theme emerged, a balancing act consisting of three categories: (1) visiting the nursing home; (2) building and maintaining relationships; and (3) gathering and conveying information. The next of kin have to balance their own responsibility for the older person’s wellbeing by taking part in their care and their need to leave the responsibility to the staff due to critical health conditions. The next of kin wanted to participate in care meetings and conversations, not only in practical issues. The findings indicate the need to improve the next of kin’s participation in the care as an equal partner. Increased knowledge about palliative care and decision-making of limiting life-prolonging treatment may lead to a higher quality of care.

## 1. Introduction

Assisting an older person in moving into a nursing home is a challenge for the next of kin [1,2,3]. It often involves mixed feelings of guilt, relief, and remorse [4,5]. In the nursing home setting, the relationships between the elderly and their next of kin may alter to a great extent [6]. Not only the move in itself, but also the continually declining health of the older person contributes to this change [7].

The current policy in the Swedish welfare system for caring for older people is “Aging in place”, which means that older people remain living in their own homes for as long as possible, even when they are in need of health care due to illness and multi-morbidity. The view behind this policy is that older people prefer this care model as it enables them to maintain their independence, autonomy, and connection with their family and friends [8,9]. It is also favored by policymakers since this policy avoids the costly option of institutional care [10]. According to “aging in place”, the Swedish policy is applied even in nursing homes where old people live in a small apartment with their own leasing contract. The right to an apartment in a nursing home is based on the older person’s need for everyday care around the clock. This typically happens when the resident is too ill and frail to continue living in their own home or when there are no next of kin available to be a care provider [2,11]. Forty-nine percent of all people over 64 years of age who died in Sweden during 2016 were living in a nursing or group home [12]. This means that nursing homes are a workplace for assistant nurses and nurses involved in the care of the dying.

The literature shows that most of the care in nursing homes is palliative in nature. Research on palliative care in nursing homes is often descriptive and there is great interest in applying palliative care principles. However, there are relatively few published studies on palliative care interventions in a nursing home setting [13]. It is also important to focus on next of kin satisfaction with the care offered in nursing homes as they fill an important role for older persons since they participate in or are sometimes needed to take over the decision-making process when the older person’s health deteriorates. After the person has died, they will live on with the memories. Next of kin participation in their care can make it easier for the physician to know if the wish of the next of kin is not to pursue a pointless extension of life or if the opposite is the case, i.e., the older person wishes to live as long as possible [14]. Studies show that resuscitation and hospitalization for older relatives are the issues most discussed, often shortly after admission to a nursing home. This generally results in a do-not-resuscitate and a do-not-hospitalize agreement [13,14]. 

The implementation of palliative care in nursing homes to a higher degree can be difficult because few next of kin are familiar with the term palliative care, which can be seen in a descriptive study about the use of an Advanced Care Plan (ACP) with 20 next of kin to people with dementia [14]. ACP is a process of discussion between the older person, the next of kin, the physician, the registered nurse, and assistant nurse in order to ensure the families’ wishes and preferences are taken into account. However, ACP is rarely used in nursing homes, and decision-making in palliative care may therefore lead to conflict between the staff and next of kin [15]. Another barrier causing delayed decisions regarding treatment and care are the different views within the family or between the family and the physician and other staff [14]. 

A considerable barrier is the lack of education provided for professionals regarding knowledge and training in the principals of palliative care [16]. Recognition of this barrier provided the impetus for the present research project, which involved the provision of educational intervention concerning knowledge-based palliative care for nursing home staff and managers. The intervention consisted of five seminars over six months for staff and managers at 20 nursing homes in two counties in Sweden [11]. The intervention was evaluated using a non-randomized experimental design with intervention and control groups as well as pre- and post-assessments [11,17]. This study provided a baseline investigation before the implementation of the principles of palliative care in nursing homes by means of the educational intervention, with the intention of achieving the best possible palliative care while at the same time involving the older persons and their next of kin in the care process. Thus, this study explored the next of kin’s experiences participating in the care of older persons residing in nursing homes.

## 2. Materials and Methods

### 2.1. Study Design

The study consisted of an explorative qualitative design based on semi-structured interviews with the next of kin, which were analyzed using qualitative content analysis [18,19].

### 2.2. Sample

The next of kin were selected from two counties in the south of Sweden that are involved in the Implementation of Knowledge-Based Palliative Care for Frail Older Persons in Nursing Homes project (acronym: KUPA project from Swedish: KUnskapsbaserad PAlliativ vård [11]). The nursing homes consisted of both larger (≥100 older persons) and smaller (<25 older persons) nursing homes situated in both urban and rural areas in both counties. The participants of this study were recruited from 20 nursing homes in each of the two counties. The distribution of participants per nursing home was related to the size of the accommodation, from four participants in a large nursing home to one participant in a small one. The inclusion criteria were to be a next of kin to an older person living in a nursing home who often visited that person, and to be able to speak and understand Swedish. Forty next of kin were invited to participate in the study and all chose to participate in the interview. The contact person primarily asked those next of kin who frequently visited the nursing home and had a close relation with the older person.

### 2.3. The Procedure of the Data Collection

The next of kin were selected by a contact person (a nurse assistant, a manager, or an administrator) at each of the nursing homes according to the inclusion criteria stated above. The contact person informed the next of kin about the study and then asked if they were interested in participating. If positive, the contact person passed on the name and telephone number of the participant to the researchers, who contacted them by telephone informing them once again about the study and asking them to confirm their interest in participating. Following this, a time and place for an interview were determined according to the next of kin’s wishes. Before the interview began, oral and written information about the study was offered once again and written consent was provided. The interviews were performed at a place chosen by the next of kin (e.g., a conference room at the nursing home or in the next of kin’s private home).

### 2.4. Interviews with the Next of Kin

The interviews were performed in a similar manner by four researchers, all of whom were registered nurses with a long experience of working with older persons as well as conducting research interviews. The interviews were based on a semi-structured interview guide about the ways the next of kin participated in the care, how they would like to participate, if they experienced any obstacles for participation, and what information they had received about palliative care (only asked if there was relevance for this question). These questions were related to the focus in the KUPA project described earlier.

After the opening question, “In what ways do you participate in the care of your relative at the nursing home?”, follow-up questions were asked. The number and formulation of the follow-up questions depended on the richness of the participant’s initial answers, and were expressed as: “Could you tell me more about your experiences?”, “What did you think in that situation?”, and “What did you do in that situation?” The interviews were conducted from April 2015 to May 2016 and ranged between 8 and 77 min (median 41 and mean 42 min). All interviews were audio recorded and transcribed verbatim.

### 2.5. Data Analysis

The data were analyzed using qualitative content analysis, which is a method used to identify categories and themes through a systematic process of coding data based on an interpretation of the content of a text [19]. A stepwise methodological approach described by Graneheim and Lundman [18] was applied, using NVIVO 11 [20] to code the data. The analysis was conducted by the first and second authors (Birgitta Wallerstedt, Lina Behm) The first step was to read the transcribed interviews several times in order to obtain an overall impression of its content regarding participation. In the second step, meaning units were identified; in the third step, they were condensed into shorter units in NVIVO (QSR International Pty Ltd, Doncaster, Victoria, Australia). In the fourth step, a preliminary interpretation of the underlying meaning was expressed in terms of codes. These steps were conducted separately by the first (Birgitta Wallerstedt) and second authors (Lina Behm). with 20 interviews each. In the fifth step, the two authors discussed together and sorted all of the codes into tentative sub-categories and categories related to the aim. In the sixth step, all authors discussed the tentative theme and categories until consensus was reached and the theme, categories, and sub-categories were established (Table 1). Selected quotations were used to illustrate the findings. The COREQ (COnsolidated criteria for REporting Qualitative research) 32-item checklist [21] was used as a guide for the reporting of the study.

### 2.6. Ethical Considerations

This study is part of the KUPA project approved by the Regional Ethics Review Board in Lund, Sweden (No. 2015/69), and with trial registration: NCT02708498. The research project is guided by the ethical principles for medical research (the Declaration of Helsinki). The participants’ confidentiality was taken into account when reporting the findings that had been done at a group level. Information related to the next of kin having the right to withdraw from the study at any time without suffering any consequences was given before each interview and written informed consent was received from each participant.

## 3. Results

The participation by the next of kin in the care of the old person in a nursing home was presented as one theme, three categories, and nine sub-categories. An overarching theme emerged from the analysis: a balancing act. This theme describes how the next of kin tried to balance their own sense of responsibility for the old person’s wellbeing by taking part in the care with their need to also leave the responsibility to the staff. The three categories comprising the theme are: visiting at the nursing home, building and maintaining relationships, and gathering and conveying information (Table 2).

Many of the next of kin described their participation in care as a routine, often having been involved in different ways for many years when the person was still living at home. Even as a routine, they had to balance their engagement in relation to their other duties and tasks in daily life. Some expressed that they wanted to participate in the care processes because of their close relation to the older person; others indicated that they did not participate at all. However, the majority were involved in the care of the old person in some way. 

### 3.1. Visiting the Nursing Home

One practical effort was to visit the nursing home. The visits were expressed as more or less frequent, alone, or together with the other parent, siblings, children, or grandchildren. Full-time work or a long distance to travel to the nursing home as well as eventual personal disability were factors that affected the possibility of visiting the old person. Other participants noted that it was all about how to prioritize. 

The visits had both positive and negative aspects. It was satisfying to talk to and socialize with the old person and make sure that they felt satisfied, but there was also concern over the old person’s condition and the feelings in relation to the situation, e.g., their own sadness over a deteriorating state.

And she remembers us and is very pleased when we visit which in some ways helps one to cope and helps provides the energy required to make the effort to visit her. Sometimes one can feel quite exhausted when it is time to leave (Daughter, 57 years).

#### 3.1.1. Helping with Practicalities

An important reason for participating in the care was to make sure that everything worked well for the old person in the nursing home. One participant compared it to having two jobs at the same time. If it was impossible for the next of kin to visit, some engaged others to replace them. During a visit, the next of kin could help with, for example, pushing the older person’s wheel-chair to the dining room, feeding, or to take the old person outside for some fresh air. Another practicality was being able to transport the old person to different appointments such as a visit to the doctor or dentist. Several next of kin used their own car to provide more comfort. Often older people feel more secure when being accompanied to outside appointments.

In the last few years I have been following my mother to every doctor’s visit. She sees and hears badly and I have supported her and have been acting as her “memory” too. Then I have to drive her by car to and from visits when she has been able to walk with walker (Daughter, 68 years).

#### 3.1.2. Helping with Private Matters

The next of kin also participated in matters of hygiene such as helping the old person to the toilet or to take a shower. Other ways of participation were doing the older person’s laundry and buying clothes for them, and further handling other private things where necessary, sometimes together with the old person. Some next of kin took responsibility for the older person’s paperwork, finances, and contacts with authorities. Visits to the bank could be accomplished with the help of a next of kin, for errands such as preparing their will. Cleaning the room was sometimes seen as a responsibility for the participants who also made the effort to make the room as home-like as possible.

They have a lot of photographs and pictures on the walls and shelves. And I…I had said to the staff at an early stage that we have no requirement towards them that they should dust or clean these items; we will do that ourselves (Daughter, 68 years).

#### 3.1.3. Enabling Activity

Some next of kin participated in social gatherings together with the old person on a regular basis but also suggested and arranged their own activities at the nursing home. To take the older person to their home or that of one of their children is an excellent way to reduce the risk that the old person should be institutionalized. For example, being able to visit a church could be very important for some residents. Another way to participate was to take responsibility for the more frequent training of the old person’s remaining abilities, for example, walking, when this was not organized by the staff. One next of kin brought an exercise bike to a nursing home where no rehab training was offered.

When father came here, he came directly from rehab after he had been fitted with his first prosthesis. They had trained him enough so that he could walk quite well. He should train continually to keep him going. However, no training was available here so he lost his ability (Daughter, 68 years).

#### 3.1.4. Controlling and Supervising the Care

Knowing that help, support, and nutritious food were offered at the nursing home around the clock and that acute situations were handled without delay created a sense of security for everyone. It required the staff’s sensitivity, understanding, and knowledge about the person. However, many next of kin found it necessary to participate in the care in a controlling function when they questioned whether the old persons’ needs were being met. Earlier troubles and irritation surrounding caregiving increased the engagement by the next of kin and the need for control, which was sometimes due to inexperienced or ignorant staff. The large number of staff involved in the care and the low trust and confidence in the staff by the next of kin (because of missed care visits) increased their desire to take control.

This was the reason why I was there those days. I was suspicious that the staff were not able feeding her properly (Daughter, 67 years).

The next of kin’s need to control resulted in their making more frequent visits to the nursing home, and sometimes they had to advocate care initiatives and be the one that made things happen.

But if I had not seen what was happening to her she would have died, of that I am sure. The staff had not noticed and the nurses are never there so I had to ask the night staff to be kind enough to take her temperature etc. during the coming night. They would not have done it otherwise. That demonstrates not following up situations that arise (Son, 64 years).

Phone calls to a next of kin during the night from a confused and frightened family member living at the nursing home also illuminated the need for control, since this situation arose when staff did not respond to the alarm. The next of kin had to support and calm down the old person until the staff finally arrived. A more responsible care of the old person was instigated following pressure from frustrated and disappointed next of kin.

Inexperienced staff who lacked knowledge about caring for older persons and unengaged physicians were perceived as a concern. The staff’s lack of action regarding the implementation of necessary changes in care, disrespect for persons’ wishes, and missing personal belongings at the nursing home, coupled to the cleaning, were also noted by next of kin. The need for improvements in the older person’s accommodation increased the next of kin’s desire for better control, and enabled some next of kin to argue their point and question the staff’s methods. Such situations created a need to intervene and to speak up and act on behalf of the older people to ensure their wellbeing, as they were not always able to complain themselves. At the same time, even though it may be difficult to question the staff’s behavior without sufficient background knowledge, it is important to have the courage to do so. In some difficult cases, the next of kin wanted to remove the person from the nursing home to a hospital in order to get the best care.

### 3.2. Building and Maintaining Relationships

When participating in the old person’s care, the next of kin became part of both a care and a private relationship. Several visits to the nursing home per week gave the next of kin time to talk to the older person about other things than, for example, symptoms or wellbeing. It could be important to tell about what was happening outside the nursing home or just to socialize without needing to do so much, just be with each other. This was explained as being very satisfying for both parts. The visit often gave the parties the opportunity to look and talk about photos on a tablet computer or in a photo album, or simply to read books or newspapers together.

#### 3.2.1. The Care Relationship 

The next of kin wanted the best care for the old person, with the opportunity to be as autonomous as possible. Being part of a care relationship was one way for the next of kin to also be involved in the care. It was important to have quality in the care relationship in terms of of how they as next of kin were treated, what possibilities there were for cooperation, and that their participation was regulated by their own wishes. Many next of kin experienced that their visits to the nursing home were appreciated by the staff and that the staff also cared about them. Being asked by the staff about their own wellbeing was especially important during the end of life phase, and being validated increased their energy and desire to participate in the care. However, in disrespectful care situations where the old person might lose their dignity, the next of kin complained and spoke to the staff on their behalf.

There were hints from the staff that she was faking or that she did it on purpose to be a bit difficult, then I felt the need to shout out and said, “She is not being difficult it is just that she is unable to do it” (Daughter, 67 years).

According to the participating next of kin, being a resource to the care team for the old person, for example, by helping with their hygiene and feeding them—was appreciated by the staff. Their involvement in the care was described as almost being a member of the staff group. It also meant that others could participate in the way they wanted to, even if they did not manage all of the practical things.

In practice I did not need to take care of her as the staff did that. Rather I was simply present.

It was good to have the staff otherwise I would need to be more involved in the practical. Yes, the purpose of my participation was to be there, to be close to her (Daughter, 67 years).

Responsibilities could also be shared between the staff and the next of kin, e.g., for washing clothes. Sometimes the next of kin needed to act as intermediaries between the old persons and the staff in order to obtain the required help or to organize a meeting concerning the old person’s wellbeing. In other cases, no cooperation from the staff was offered at all; instead, the next of kin were obliged to carry out the staff’s duties as the staff were elsewhere doing other things.

I don’t really think I am participating in anything more than being involved in her as a person. Definitely nothing in her care. I don’t feel that kind of cooperation exists at all down there (Daughter, 62 years).

#### 3.2.2. The Private Relationship

The private relationship between the next of kin and the old person as well as with other next of kin meant closeness, support, joy, natural togetherness, reciprocity, and being a lifeline, which are the conditions for a care relationship. It was seen as as a point of security that the old person now lived in a nursing home. However, illness and deterioration partly changed the relationship and limited private dialogue, implying a risk of loneliness and exposure on the part of the older person according to the next of kin. A conversation could concern everyday events, but also more difficult topics such as the matter of the eventual disposal of the older person’s property. The old person’s difficulty in speaking up about their own wishes and needs to the staff could be another matter for discussion.

The staff are not mind readers so one must speak up. They cannot know what she wants if she doesn’t tell them and it doesn’t help to tell me as I am only here once a week. (Daughter, 68 years).

Sometimes the old person’s present life situation was experienced by the next of kin as being sad and not a real life. This promoted worry with feelings of disappointment and anger. Furthermore, feelings of guilt arose for not being with the old person as much as they ought to or because they felt fit while the old person deteriorated. The old person’s wishes at the end of their life was also a hard topic to talk about and almost none of the next of kin had succeeded in doing that. Neither the next of kin nor the old person really wanted to talk about that, even though the old person was deteriorating.

I don’t know! I almost said that I don’t wish to imagine it. We have talked so much about it so I know that my father has chosen a place at the cemetery where he would like to be interred. It also came up a while back (Daughter, 68 years).

One reason for the difficulty of talking about this topic was that it could be interpreted as wishing away someone’s life. It seemed to be more manageable to talk with the old person about their wishes concerning their funeral than dying and death.

According to the next of kin, participation in the care of the older person at this stage in life was important to consider and a shared responsibility for the care was crucial for their participation. They felt it important to have symptom relief available and that the older person did not have to die alone.

#### 3.2.3. Adopting to New Roles 

In their participation in the person’s care, the next of kin talked about taking responsibility and the need to prioritize. Relinquishing responsibilities was not easy and some next of kin struggled with changing priorities. Some next of kin thought they still had the overarching responsibility for the old person. They continued to be engaged even if they did not need to because they were used to taking the old person into account. However, since this person was now living in a nursing home the situation was more relaxed. Other next of kin accepted that the staff had taken over the responsibility for caring and their task was to make sure that the old person was doing well and to offer a silver lining to their life. However, some next of kin could not leave the responsibility to the staff but felt it necessary to be available in case something untoward should happen to the old person.

Some next of kin who took their vacations still visited regularly, others thought they did not need to. There were also opinions that the nursing home care was safe; therefore, there was no need to worry to check or take special responsibility. Still, the old person was always on their mind. Others argued that they as next of kin should have a life of their own without so much responsibility. It was also noted that the responsibility for the old person was often divided among the family, for example, among siblings, which created an imbalance as often one took more responsibility than the others.

I have two brothers and a sister and I kind of felt that it is mostly I who had to take the responsibility. Yes, it did seem that way and she lived a little closer to me than the others. Yes, it was tough but then she moved to the nursing home so now everything functions very well (Daughter, 62 years).

The reason for such an imbalance was that the next of kin all had different personalities, qualifications, depths of engagement, and time available, although all tried to do their best. It was also said that the level of responsibility varied between men and women and that age was an important factor for the level of engagement. Sickness on the part of a next of kin could prevent their participation in the care. However, in some way or another, the responsibility for visits and tasks was divided up among them and thereby they complemented each other. Overall, it was noted that if the next of kin had not participated to the degree they did, a contact person or a legally appointed guardian would be obliged to take on the responsibility for the older person. Leaving the responsibility for the old person’s care to the nursing home staff made it possible for the next of kin to run their daily lives in another way. Some next of kin had learnt through experiences in life and training that it was not possible to be engaged in all matters, and that you had to leave some. To have a choice was described as a relief, but a prerequisite was the trust that the older person received adequate help and was cared for with dignity.

### 3.3. Gathering and Conveying Information

Contact with the staff and the quality of information that the next of kin received had an impact on both the care and private relationships with the old person as well as the next of kin’s participation in care. Basic information was usually given at the time the older person moved to the nursing home.

#### 3.3.1. Having a Dialogue with the Staff

Prerequisites for developing a continuous dialogue concerning the care of the elderly included the staff’s interest and openness to take the time to learn the older person’s background history, their needs, and wishes, in addition to those of the next of kin. A key person to keep the dialogue alive was the contact person. Furthermore, a notebook placed in the room was also important for making notes and sharing information related to the older person.

He and I and his carer talk all the time. So we sort of have a dialog on what’s going on all the time which I find important. I can’t really just come here and say “Hi”, talk for a bit, then go home. I want to be a part of things (Son, 51 years).

Sometimes communication just happened, perhaps over a cup of coffee or via a text message. It was highly important for the next of kin to be invited to care meetings at the nursing home. 

Additionally important was the ability to receive information about what had taken place since their last visit to the older person. The possibility to ask questions about the current situation and communicate information about and together with the old person was valuable. The management of delusions or deterioration could increase the need for dialogue and closer contact with the staff via more frequent visits and communication.

For example, information about deterioration could be difficult to receive, but it was important to understand that in such a situation it was necessary for a next of kin to visit the old person without delay. Also, it was essential to communicate correct information to other next of kin about the actual situation. In some cases, discussions about resuscitation or other active management to prolong life took place. Routines concerning dying and death at the nursing home was sometimes conveyed to the next of kin, but almost none of them had received any background information concerning palliative care and its meaning.

There was a deterioration and I thought she had perhaps sustained a small blood clot or something like that. Then they rang and talked, first they talked here and then they rang and talked to me. So I feel that I was involved. That is to say, as much as was possible (Daughter, 57 years)

Sometimes the dialogue about the old person’s wellbeing was scant. The next of kin did not talk much to the staff, and the staff did not ask the next of kin any questions or request their opinions. Lacking communication within the organization and between the care levels concerning the care of the older person was highlighted. Some of the next of kin had requested a visit from a physician for the old person, but had received no answer about when that would happen, so they gave up attempting to get in touch with a physician. In some cases, the next of kin had not been invited to meetings involving follow-up of the care, and decisions were made without either the older person or the next of kin being involved.

#### 3.3.2. Calling and Writing Letters

Using telephone calls to ask for information as an alternative to visiting the nursing home would give the next of kin the possibility to be involved in the old person’s life and care.

If I do not have the opportunity to come here, I always ask how she is. It is very rare that someone has said “X is not so good” rather they reply that she is well. Then, as a rule, I take the opportunity to also talk to her (Daughter, 57 years).

If the ability to communicate in this way with the old person was lost, it became difficult for the next of kin to know and understand the old person’s actual situation, which raised the question whether there was an urgent need to visit the old person. Sometimes letters were used to communicate, for example, when it was hard to visit the old person due to distance, own disability, or relationship problems. 

## 4. Discussion

This study sought to explore how the next of kin experienced their participation in the care of older persons (family members) at nursing homes. An overarching theme emerged, that of a balancing act, which concerns the balancing of, on the one hand, wanting to maintain and, on the other, wanting to let go of their responsibility for the old person’s care. This balancing act was managed by means of visiting the nursing home, building and maintaining relationships, and gathering and conveying information. 

The overarching theme of the balancing act reflects the next of kin’s ambivalence towards the responsibility for the care and wellbeing of the older person. Other studies have described this in terms of mixed feelings. For example, Eika et al. [3] noted that the next of kin of an older family member who had moved to a nursing home in Norway were relieved, but also felt apprehensive about the quality of care the older person received. Similar to our findings, they observed that many next of kin continued feeling responsible for the older person, although they recognized that they were in a critical condition and there was need for nursing home care. In an ethnographic study of a nursing home ward in Sweden, Whitaker [7] concluded that the motives for involvement in the older person’s life consisted of, on the one hand, love, responsibility, obligation, and repayment, and on the other hand, guilt and a bad conscience.

The balancing act by the next of kin may be understood in terms of attitudinal ambivalence, a concept introduced by Scott [22], which has been defined by Gardner as a psychological state in which “a person holds mixed feelings (positive and negative) towards some psychological object” [23]. Hence, attitudes are said to be ambivalent when something such as the responsibility for the older person’s care is evaluated both positively and negatively at the same time [24]. Research has shown that higher levels of attitudinal ambivalence create discomfort, which leads people to seek out consensus information in order to solve the conflict and reduce dissonance [25]. People are also more open to persuasion when they experience high attitudinal ambivalence toward the target object [26]. This points to the importance of nursing home staff reassuring the next of kin of the quality of the care and allowing the next of kin to become involved to the extent they wish. Eika et al. [3] emphasized the importance of the next of kin and the older person feeling appreciated in the nursing home environment and of the feeling of being at home in the nursing home [27].

We found that the visits concerned both more practical day-to-day care (i.e., instrumental care) and care that served more social and emotional purposes (i.e., non-instrumental care) as expressed in the category of “Visiting the nursing home” and the four sub-categories of “Helping with practicalities”, “Helping with private matters”, “Enabling activity”, and “Controlling and supervising the care”. In a study of family involvement in nursing home care in Australia, Irving [28] showed that the next of kin who participated in assisting with instrumental care, including activities of daily living, were less satisfied with the nursing home care and that the next of kin usually wanted to limit their level of involvement in instrumental care. Greater value was placed on the possibility of providing non-instrumental support such as advocacy, advice on the older person’s preferences, and care oversight [28]. Our study showed that the visits by the next of kin to the nursing home involved different types of activities. In addition, our findings revealed that the next of kin who were dissatisfied with the quality of care felt the need to control and supervise the care at the nursing home. Some of the next of kin in our study expressed frustration with the care provided by the staff, resulting in a lack of trust and confidence in the nursing home. Whitaker [7] observed that during visits to the nursing home, the next of kin often looked for signs of neglect or lack of proper care of the older person. This represents a form of control of the staff in order to preserve the older person’s dignity. Whitaker calls this “the impossible” role of the next of kin; they cannot always be present and constantly guard the older person’s integrity or make sure that she or he is well cared for and respected [7].

While research on family involvement in nursing home care has tended to focus on the instrumental caring role of the next of kin, Whitaker [7] argued that describing next of kin involvement according to the extent to which the next of kin carried out certain instrumental tasks was misleading and did not do justice to all of the caring activities characterized by the concern and interest in the old person’s care and wellbeing.

Viewed from a broader perspective of the next of kin’s visits in our study that concerned both instrumental and non-instrumental care, Whitaker [7] believed that the next of kin’s visits to nursing homes had both a symbolic and ritual meaning besides their practical nature. The visits reinforced the feeling that the old person was not alone, but part of a larger community beyond the nursing home. Thus, the visits may be seen to represent a promise of the continuance of life despite the nearness of death.

Our findings showed that the next of kin were anxious to maintain the relationship with the old person, i.e., the sub-category “The private relationship”. Transfer to a nursing home involves a change for both the next of kin and the older person that disrupts their previous routines, but new habits develop in the nursing home [29]. While the frequency and duration of visits by next of kin tend to vary considerably [6], our study underscored that the next of kin wanted to remain involved in the life of the older person. Whitaker [7] argued that visits to the nursing home “represent” the relationship, regardless of whether it was a parent-child relationship, friendship, or marital relationship. Although the relationship depends on the physical and mental condition of the older person, other studies have confirmed the importance of the relational links outside the nursing home [30,31]. 

The relationship between the next of kin and the nursing home staff was also important, as seen in our sub-category “The care relationship”. We found that the next of kin, in general, felt appreciated by the staff, but other studies have suggested that this relationship could be somewhat difficult. For example, Eika et al. [3] noted that many of the next of kin had little communication with the nursing home staff and some felt that they disturbed the nursing home’s routines. The next of kin who perceived that they had limited support from the nursing home staff were unsure about what to expect from the staff.

It is important that the nursing home staff are aware of and are able to support the next of kin’s process of adapting to new roles after the older person’s move to a nursing home, which represents a profound life transition for both the older person and their next of kin. They continue to be involved and struggle to adjust their relationship with the older person and establish new roles [3,30]. Many of the next of kin in our study appeared to have trouble letting go of their responsibility for the older person’s care, but others argued that the responsibility had now been taken over by the nursing home. The importance of the roles that emerge when an older person moves to a nursing home has been increasingly emphasized in the research [7]. The literature describes a wide range of roles played by the next of kin [32,33].

The next of kin’s need for participation in the older persons care was additionally illustrated in our findings under the category “Gathering and conveying information”. Phone calls and email were used when the next of kin had no possibility of visiting the older person due to other duties or when they could no longer talk with the older person due to dementia. This became a way to follow the health condition of the older person every day, but the most valuable support perceived by the next of kin was when they were invited to participate in care meetings and had the possibility to ask questions about things such as illness deterioration. The next of kin expected staff to recognize their need for information and guidance in their involvement; however, they often felt that this guidance was lacking [34]. Furthermore, many of the next of kin argued that they were not invited to be equal partners in decisions about the care [35]. Furthermore, the next of kin were sometimes afraid of being perceived as troublemakers and therefore many of them did not ask too much or complain too much about the staff [36]. 

The interviews with the next of kin in this study were made before the implementation of knowledge-based palliative care through an educational intervention for the staff [11]. With staff that have only a limited knowledge about palliative care, it is not surprising that communication with the next of kin about palliative care was unusual. However, it would have been expected that they were invited more frequently by the staff to take part in the care of the older person. Insufficient participation by the next of kin was also found in an interview study from Norway with the next of kin that took place 2–12 months after the death of a patient, concerning their involvement and experiences of decision-making processes related to the issues of limiting life-prolonging treatment [37]. Their results showed distinct shortcomings regarding the next of kin’s participation in decisions on life-prolonging treatment in nursing homes. The staff only first contacted the next of kin for a discussion when the patient’s condition had deteriorated and their life was approaching its end. If conversations did take place early in the process, this was a result of the next of kin having asked for a dialogue [37]. Most relatives wanted to be involved in the decision-making process, as well as to receive information about the patient’s health condition. The next of kin wanted the staff to initiate the conversation about these issues since they felt they were emotionally challenging [38]. Research has described communication about the end of life as a challenge for nursing home staff [39] and revealed the need for education and training for this task [40,41]. However, the crucial point was that the benefits for the next of kin of using a palliative approach of care [42] in nursing homes for old persons must be fully understood [43,44].

Choosing participants with a diversity of experiences enhanced the credibility of this study [18,45]. Interviews with 40 next of kin in two counties in Sweden and from 20 nursing homes, large as well as small, situated in both urban and rural areas, were the procedures undertaken to attain credibility and transferability of the results. Therefore, the next of kin in this study represented nursing homes providing varying qualities of care and had different cultures, norms, and organization sizes. This indicates that our findings may have better transferability to other nursing homes compared to a study in which the interviewees were few and selected from only one nursing home. However, the limitations were that none of the next of kin were younger than 40 years and that we did not know the level of palliative care or end-of-life care that their older relative received when the interviews were made.

The use of four different researchers to conduct the interviews may have had a negative effect on the credibility of the findings. On the other hand, they were all nurses with a long experience of aged care. The fact that two of the researchers who conducted the interviews also analyzed the text independent of each other and had several meetings together concerning the interpretation of the texts, i.e., investigator triangulation, may have conferred a positive effect. In addition, the analyses were scrutinized by all of those in the research group to manage the bias embedded in close engagement. For the sake of credibility, the analytical process has been carefully described so the reader may follow the researchers’ interpretations. The interviews served as a point of reference throughout the analytical process when a deeper understanding was required of the meaning units, codes, and categories. Hence, to make the results more credible, the quotations represent different next of kin and different relationships with older persons.

## 5. Conclusions

Being a next of kin of an older person in a nursing home involves an act of balance between keeping the overall responsibility for the older persons’ care themselves, or trusting the nursing home staff and leaving the responsibility for the care to them. The findings confirmed previous research that the next of kin were anxious to be engaged in how the care at the nursing homes was performed. They wanted to participate in care meetings, and not only in practical tasks; however, they considered their practical participation in the daily care as an important dimension for the wellbeing of their loved ones under care. The attitudes and culture of the staff in welcoming the next of kin as equal partners needs to be supported by education as well as increased knowledge about palliative care and decision-making related to limiting life-prolonging treatment.

## Figures and Tables

**Table 1:** Characteristics of the participating next of kin (*n* = 40).

Characteristics	No.	%
**Age, years**		
40–49	1	2.5
50–59	11	27.5
60–69	18	45
70–79	8	20
80–89	2	5
**Gender**		
Men	10	25
Women	30	75
**Marital status**		
Married/living together	32	80
Unmarried/divorced	6	15
Widower/widow	2	5
**Relation to the old person**		
Husband/wife	7	17.5
Daughter/son	31	77.5
Sibling	1	2.5
Other	1	2.5
Educational level *		
Elementary school	9	25
High school	8	22
Trade school	4	11
University/college	15	42
**Work status**		
Full time	13	32.5
Part time	9	22.5
Not working	18	45
**The frequency of visits to the old person**		
Every day	6	15
Weekly	31	77.5
Monthly	2	5
Yearly	1	2.5

* Four people were missing this information.

**Table 2:** The result presented in theme, categories, and sub-categories.

Theme	Category	Sub-Category
The balancing act (between having and leaving responsibility)	Visiting the nursing home	Helping with practicalities
		Helping with private matters
		Enabling activity
		Controlling and supervising the care
	Building and maintaining relationships	The care relationship
		The private relationship
		Adapting to new roles
	Gathering and conveying information	Having dialogue with the staff
		Calling and writing letters
